# Socio-Demographic Correlates of Basic Food Needs: A Maslow’s Hierarchy Analysis

**DOI:** 10.3390/foods15010057

**Published:** 2025-12-24

**Authors:** Nicoleta Defta, Andreea Barbu, Violeta Alexandra Ion, Livia Vidu, Elena Peț, Liviu-Cristian Cune, Liliana Aurelia Bădulescu

**Affiliations:** 1 Faculty of Animal Productions Engineering and Management, University of Agronomic Sciences and Veterinary Medicine of Bucharest, 59 Mărăști Blvd., 011464 Bucharest, Romania; nicoleta.defta@usamv.ro (N.D.); livia.vidu@usamv.ro (L.V.); 2 Research Center for Studies of Food Quality and Agricultural Products, University of Agronomic Sciences and Veterinary Medicine of Bucharest, 59 Marasti Blvd., 011464 Bucharest, Romania; andreea.stan@qlab.usamv.ro (A.B.); liliana.badulescu@qlab.usamv.ro (L.A.B.); 3 Faculty of Management and Rural Tourism, University of Life Science “King Mihai I” from Timișoara, 119 Aradului Road, 300645 Timișoara, Romania; elenapet@usvt.ro; 4 Department of Theoretical Physics, Horia Hulubei National Institute of Physics and Nuclear Engineering, 077125 Măgurele, Romania; 5 Faculty of Horticulture, University of Agronomic Sciences and Veterinary Medicine of Bucharest, 59 Marasti Blvd., 011464 Bucharest, Romania

**Keywords:** food purchasing behavior, cross-sectional survey, safety needs, food security, food marketing strategies

## Abstract

Nutrition is a fundamental aspect of consumer behavior, closely linked to the satisfaction of basic household needs and strategies for purchasing food products. This study aimed to examine how fundamental food needs—specifically survival (daily food) and food security (food stocks)—shape purchasing behaviors, enabling the identification of vulnerable consumer segments and the delineation of patterns useful for producers and retailers. Data were collected through a cross-sectional survey (N = 1060) and analyzed using the Rao & Scott-adjusted Pearson chi-square test (R, version 4.4.3), considering key socio-demographic factors including gender, age, educational level, marital status, residence, and income. Results indicate that gender, age, and education significantly influence food purchases driven by the need for food security, whereas marital status is a significant factor only for survival-related purchases. Differences observed in other contexts were not statistically significant. Additionally, two multinomial logistic regression models were developed to predict consumer food purchases driven by fundamental needs, demonstrating high explanatory power. Each socio-demographic factor emerged as a significant predictor for at least one response category on the Likert scale, and the relative influence of each predictor was quantified. These models provide actionable insights for marketing strategies, including the identification of optimal store locations and the adjustment, diversification, or optimization of product ranges based on the characteristics of specific consumer segments and geographic areas.

## 1. Introduction

Nutrition is one of the essential dimensions of daily life and is directly related to the satisfaction of an individual’s fundamental needs. Within Maslow’s hierarchy of needs [1,2], the first two levels—physiological needs and safety needs—form the foundation upon which higher-order motivations are built. Among these, food plays a central role: on the one hand, as a biological necessity indispensable for survival, and on the other, as a component of safety associated with reliable and predictable access to food resources. Decisions regarding food consumption cannot be fully understood outside the fundamental context of maintaining daily life and stability.

In contemporary society, characterized by accelerated urbanization, economic uncertainties, and rapid social change, food purchasing has become a direct expression of concerns related to basic needs. Beyond shopping aimed at meeting daily requirements, the trend of food stockpiling as a precautionary strategy reflects how individuals respond to risks and perceived uncertainty. This type of behavior has been particularly evident during times of crisis, such as the COVID-19 pandemic, when consumers engaged in preventive purchases— accumulating food and sanitary products—in response to anxiety about resource availability [3,4]. Psychological factors, including perceived scarcity, anxiety, uncertainty, and reduced trust in institutions, significantly predict panic buying and stockpiling behaviors during crises, highlighting the centrality of emotional and cognitive processes in food-related decisions [5,6,7,8,9].

Furthermore, post-pandemic analyses indicate that consumers’ perceptions of local food’s intrinsic (e.g., taste, health) and extrinsic (e.g., environmental impact, price) attributes strongly influence purchasing decisions. The psychological drivers of local food consumption include food security, trust in producers, and supply risk evaluation [10,11,12]. These insights highlight the importance of integrating the literature on the psychological drivers of food purchasing into conceptual frameworks for understanding consumer behavior.

However, the dimension of nutrition extends beyond the purely physiological need to encompass the aspect of security, understood as constant and stable access to adequate food. The modern concept of *food security* includes not only the availability of food resources but also their economic and social accessibility [13]. When this stability is lacking, food insecurity and increased levels of stress, anxiety, or depression are frequently observed [14,15], confirming the link between basic needs and overall well-being.

Differences in consumers’ return to pre-pandemic purchasing habits also reflect cross-cultural and socio-economic vulnerabilities. Perceptions of quality, price, sustainability, and accessibility of local foods are shaped by diverse cultural, social, and economic contexts. Empirical evidence shows that motivations and sensitivities regarding local or sustainable food vary across countries, regions, and generations, highlighting the need to consider cross-cultural consumer vulnerability in food consumption models [16,17,18].

Furthermore, the progressive satisfaction of basic needs significantly influences food consumption patterns. According to Van Lenthe et al. [19], individuals who have reached higher levels of Maslow’s hierarchy tend to adopt healthier eating habits, characterized by greater dietary diversity and increased consumption of fruits and vegetables. In contrast, survival under conditions of economic vulnerability dictates food choice, often leading to a preference for products with high caloric value and low cost [20,21].

Socio-demographic differences amplify the ways in which these needs are expressed through food consumption behaviors. Factors such as gender, age, education level, marital status, place of residence, and net income shape and influence perceptions and strategies related to purchasing food products. For example, in rural areas, direct access to agricultural resources can reduce the pressure to store food, whereas in urban areas, dependence on commercial supply chains can increase vulnerability to food insecurity [22,23].

Moreover, recent cross-national and regional studies have highlighted that sociodemographic factors strongly condition consumer vulnerability, perceived risk, and coping strategies in times of crisis. Evidence from countries in Central and Easter Europe, including Romania, indicates that income instability, household structure, and urban–rural differences are correlated with distinct changes in shopping frequency, stockpiling intensity, and food choice motives [24,25,26,27]. The decline in local food purchases after the initial waves of the COVID-19 pandemic has also been linked to the perception of shortage risk and the need to stockpile food [28]. Stockpiling behaviors, such as prioritizing nonperishable over fresh local products, reflect adaptations to perceived supply uncertainty and trust in the supply chain. These behaviors demonstrate that risk perception and trust-related factors, along with economic considerations, must be incorporated into conceptual frameworks to fully explain consumer responses [29,30].

Thus, the need for food can be viewed as a continuum—from meeting the immediate demands of survival, such as access to calories, nutrients, and hydration [1], to the dimension of food security, which reflects the desire for medium- and long-term predictability and stability [31]. This transition has major implications for food consumption decisions: while the focus at the survival stage is on quantity and accessibility, the food security stage emphasizes diversification, stockpiling, and resource protection.

Topics such as food safety and food security are frequently explored in the literature, reflecting both public health concerns and the stability of the global food system. The impact of external factors (including climate change, globalization, and pandemics) on food security has been extensively studied [32,33,34,35,36,37,38,39]. At the same time, recent research emphasizes the risks associated with the *food safety dimension*, highlighting its implications for population health [40,41,42,43,44,45,46,47,48]. The coronavirus disease 2019 (COVID-19) pandemic has intensified these concerns, generating a surge of studies on food insecurity and associated social vulnerabilities [49,50,51].

In addition, a significant part of the literature has focused on the conceptual clarification of food security and nutrition, offering increasingly refined theoretical frameworks and measurement indicators [52,53]. However, despite the breadth of previous academic contributions, most studies primarily address macroeconomic, health [54], or public policy dimensions [55,56], insufficiently integrating the psychological, motivational, and behavioral mechanisms that drive food purchasing decisions. Recent literature demonstrates that perceived threat, scarcity cues, loss aversion, and emotional distress are central predictors of food purchasing and stockpiling [7,57]; however, these constructs are rarely examined in relation to basic motivational needs or sociodemographic variability.

Furthermore, although several studies use Maslow’s hierarchy as a conceptual reference, the theory is seldom operationalized to explain concrete food purchasing behaviors. For example, Sobaih & Moustafa (2022) [8] showed that perceived crisis and anxiety influence excessive food purchasing. However, the interplay between motivational needs, sociodemographic differences, psychological drives, cross-cultural vulnerability, and risk perception remains largely unexplored. This highlights a substantial theoretical gap: contemporary literature lacks a coherent framework linking fundamental needs (survival and safety), psychological drives, cross-cultural variability, and risk perception to food purchasing, stockpiling, and food security behaviors.

Building on these considerations, this study investigates the role of fundamental needs—survival and food security—in shaping food purchasing decisions, based on Maslow’s hierarchy of needs. The analysis examines how sociodemographic variables influence these behaviors, highlighting differences and specific characteristics across sociodemographic categories. This study integrates motivational theory with recent insights on psychological drivers, cross-cultural vulnerability, and risk-related behaviors to provide a more robust conceptual and empirical understanding of the mechanisms through which individuals perceive and respond to threats to basic food needs.

This approach enables a more nuanced understanding of food consumption and offers valuable guidance for developing policies and strategies aimed at strengthening individual and community food security. The findings of this research can also provide evidence to guide marketing and communication strategies for producers and retailers, enabling them to tailor their approaches to consumers’ sociodemographic characteristics. Such an orientation not only enhances the effectiveness of commercial messages and interventions but also promotes a responsible approach toward vulnerable population segments from the perspective of food security.

## 2. Materials and Methods

### 2.1. Participants, Recruitment, and Procedure

This study is part of a larger research that uses a cross-sectional online questionnaire conducted between October 2021 and June 2022 to explore sociodemographic factors, segmentations, and predictors of various consumer habits, perceptions, and food needs. The same questionnaire was previously used to analyze sociodemographic factors in consumer perceptions of food promotions [23] and preferences of vegetable chips [58].

In this article, we report the results concerning sociodemographic factors and predictors related to fundamental needs from Maslow hierarchy—namely, survival (daily food) and food security (food stocks). In Maslow’s hierarchy, the need for survival through daily food belongs to the foundational physiological level, where immediate nourishment is essential to sustain life (e.g., eating a simple meal today, drinking clean water, or consuming enough calories). By contrast, the need for food security is situated at the safety level, reflecting the assurance that food will remain reliably available in the future (e.g., maintaining a pantry with staple foods, having reliable access to supermarkets or local markets, or storing frozen or preserved foods).

While daily food addresses the urgent requirement to eat today, food security provides stability and freedom from anxiety about hunger tomorrow, enabling individuals to progress toward higher-order needs such as belonging, esteem, and self-actualization. It is worth emphasizing that participation in the questionnaire was voluntary and uncompensated. All participants were informed about the purpose of the research and had the option to withdraw at any time.

The questionnaire was divided into two sections. The first section collected information on sociodemographic factors, including participants’ gender, marital status, education level, area of residence (urban or rural), and income. The second section focused on consumer behaviors and perceptions related to health, dried vegetable consumption, chip preferences, [58] perceptions of food promotions, and various food needs—including the fundamental needs of *survival* and *food security*.

The key items relevant to this study assessed the extent to which each of these needs influences food purchasing decisions, using a five-point Likert scale ranging from *strongly disagree* to *strongly agree*. This scale enabled the quantification of responses and the identification of the degree to which consumers based their food purchasing decisions on fundamental needs. The specific item was formulated as follows: “*When you purchase food products, do you do so out of: (a) the need of survival need (daily food); (b) the need for food security (food stock)?*”. Respondents selected one of the five options: *strongly disagree, disagree, neutral, agree,* or *strongly agree*. Other items followed a similar format.

The questionnaire was administered to a sample of 1060 participants, including both men and women from various sociodemographic groups. This sample size is considered substantial for the Romanian population. To better reflect the adult population of Romania (aged 18 and over), a weighted survey design was constructed using the *svydesign* function from the R *survey* package (version 4.4.3) [59,60]. The weights were derived from official national statistics [61,62]. Sample proportions were adjusted for all sociodemographic factors included in the questionnaire, except for income, for which the national data did not provide the required level of detail. The final design yielded a weighted total of 1748 and an average total variation distance of 0.18, indicating that only 18% of the sample deviated from perfect representativeness.

Although the Likert scale provides a standardized measure of respondents’ attitude and opinions that is easy to administer and compare, it has well-known limitations. Respondents tend to avoid the extreme categories of the scale, and the format may fail to capture the full complexity of real attitudes. In addition, voluntary online surveys are subject to bias-related limitations [63]. Self-selection bias can occur even without explicit invectives, as individuals with stronger opinions or more available time are more likely to participate. Coverage bias is relatively low in Romania, given the country’s high level of internet access. However, demographic imbalances may still arise in online samples; these were addressed using a weighted survey approach.

### 2.2. Study Objectives

This study conducts an exploratory, cross-sectional analysis of how sociodemographic factors (gender, age, education level, marital status, residence, and net income) differentiate and influence food purchasing behavior driven by basic food needs—namely, survival (daily food) and food security (food stocks). The focus is on identifying vulnerable categories in order to inform consumer segmentation and support strategies.

**O1:** To analyze the relationship between basic food needs, according to Maslow’s hierarchy, consumer segmentation, and sociodemographic characteristics, in order to identify significant differences in food purchasing behavior across gender, age, marital status, education, residence, and income categories.

**O2:** To identify sociodemographic predictors of food purchasing behavior based on the fundamental food needs described in Maslow’s hierarchy.

### 2.3. Research Questions

To achieve the objectives of this study, a research plan incorporating descriptive, confirmatory, and exploratory components was developed. Based on this plan, the following research questions were formulated:

**RQ1.** To what extent do food purchases motivated by basic food needs (survival and food security) differ significantly across sociodemographic categories, thereby providing a foundation for consumer segmentation?

**RQ2.** Which sociodemographic factors best predict the likelihood that food purchases are driven by basic food needs (survival and food security)?

**Figure 1:**
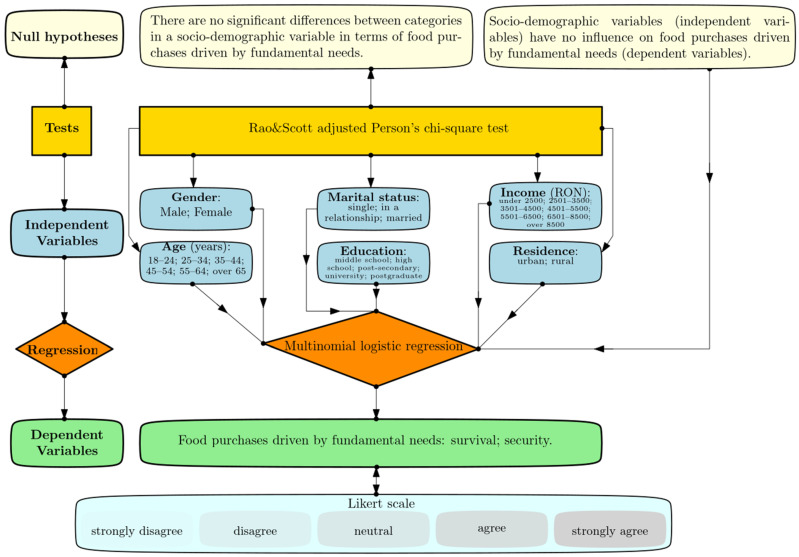
Research design flowchart.

To address these research questions, fourteen null hypotheses were tested—seven for each fundamental need), as illustrated in the flowchart (Figure 1).

The research questions and hypotheses were designed to examine how sociodemographic factors differentiate the needs for survival and food security in relation to food purchasing behavior. The objective of this study was to identify consumer segments and sociodemographic predictors of these behaviors, thereby enabling more precise consumers segmentation and the adaptation of commercial and social strategies to consumers’ actual needs.

### 2.4. Data Analysis

All statistical analyses were conducted using R version 4.4.3 [59,60,64]. Survey data were imported into R, thoroughly validated, and verified to be complete and free of missing values. A weighted survey design, representative of the adult population in Romania aged 18 and over, was constructed using the available sociodemographic data from the most recent national census [61] and the TEMPUS database [62].

The following statistical procedures were employed to test the study’s hypotheses:

(i) Pearson’s chi-square test with Rao & Scott adjustment for weighted survey designs [65,66] was applied to assess whether differences across sociodemographic categories (e.g., gender, age, marital status, education level, net income, and residence) regarding each basic food need were statistically significant. Statistical significance was determined at *p* < 0.05. It is important to note that this test provides evidence of association between variables, but only in isolation, without indicating the strength or relative proportions of these relationships. The magnitude and distribution of such effects are more appropriately examined using regression models that incorporate multiple variables.
(ii) Multinomial logistic regression analysis was performed to evaluate correlations between variables and identify significant predictors. Multinomial regression is appropriate for dependent variables representing fundamental food needs, as these have five outcome levels*: strongly disagree, disagree, neutral, agree*, and *strongly agree* (Figure 1). In contrast, binary logistic regression can handle only two outcome categories and is therefore unsuitable for this study. This method estimates the odds of each outcome relative to a reference category, which was set as the *strongly disagree* response, and provides interpretable coefficients to measure how the outcome changes with small variations in the predictor. Multinomial regression is thus effective for identifying patterns, relationships, and trends, as well as for forecasting potential outcomes.

For each independent variable, the following parameters were calculated: regression coefficients (β), standard errors (S.E.), Wald z-values, associated *p*-values (Sig.), odds ratios (Exp(β)), and confidence intervals (CI for Exp(β)). Due to the large volume of data, only the values for statistically significant predictors (*p* < 0.05) are presented. Model fit and goodness-of-fit were assessed using the McFadden’s [67], Cox & Snell’s [68], and Nagelkerke [69] pseudo-R^2^.

It should be noted that interactions between sociodemographic variables (such as age and income or education and residence) were not explicitly examined in this study, as such an analysis was beyond the scope of this article. The primary objective was to assess the independent associations between key sociodemographic factors and basic food needs. The multivariable influence on basic food needs was modeled using multinomial logistic regression.

## 3. Results

**For RQ1: Pearson’s Chi-Square Test**

To examine potential differences across of sociodemographic categories in relation to food purchases driven by the needs for survival and food security, the items used in the analysis were measured using a five-point Likert scale: *strongly disagree* (need for survival: 2.2%; need for food security: 7.6%), *disagree* (need for survival: 1.0%; need for food security: 20.4%), *neutral* (need for survival: 11.5%; need for food security: 35.8%), *agree* (need for survival: 59.7%; need for food security: 34.5%), and *strongly agree* (need for survival: 25.6%; need for food security: 1.7%).

### 3.1. The Need for Survival (Daily Food)

**Null Hypothesis H01.1.**  *There are no significant differences between gender categories in terms of food purchases driven by survival needs (daily food).*

The Rao & Scott-adjusted chi-square test revealed no significant association between gender and food purchases driven by survival needs. The test yielded F [2.13; 2254.79] = 0.36, *p* = 0.71, indicating that the observed differences across gender categories are consistent with random sampling variation. Therefore, gender does not appear to influence the likelihood of purchasing daily survival-related food items.

**Figure 2:**
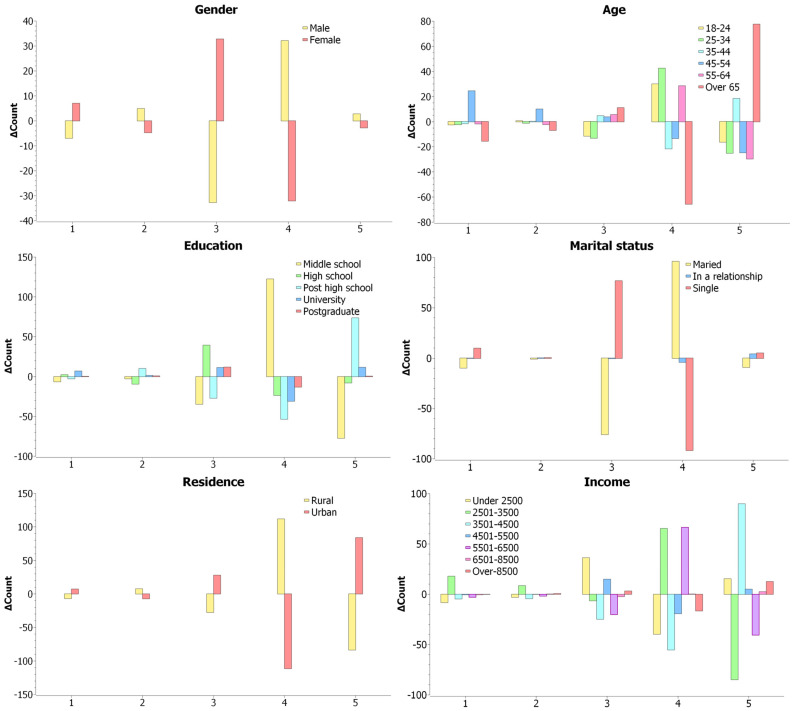
The difference between count and expected count for food purchases driven by survival needs. Notes: 1 = strongly disagree; 2 = disagree, 3 = neutral, 4 = agree, 5 = strongly agree. ∆Count > 0 indicate overrepresentation and ∆Count < 0 indicate underrepresentation.

At an interpretative level, analysis of differences between observed and expected values shows that men were overrepresented in the *agree* category (+32.1) and underrepresented in the *neutral* category (−32.8), while women presented the opposite pattern (Figure 2).

From a practical standpoint, these differences suggest that communications concerning daily food planning and preparation could be more precisely tailored to reflect these subtle behavioral preferences, even though no statistically significant gender differences are evident at the statistical level.

**Null Hypothesis H01.2.**  *There are no significant differences between age categories in terms of food purchases driven by survival needs (daily food).*

The Rao & Scott-adjusted chi-square test revealed no significant association between age category and food purchases driven by survival needs. The test resulted in F [6.51; 6895.78] = 1.23, *p* = 0.28, indicating that the observed variation across age groups does not exceed what would be expected by chance. Consequently, age does not appear to influence the likelihood of purchasing food items related to daily survival.

Descriptive analyses, however, revealed age-related patterns in food purchases driven by survival. Young adults (18–34 years) were markedly overrepresented in the *agree* category (+30.1 for 18–24 years and +42.5 for 25–34 years) but underrepresented in *strongly agree* (16.4 for 18–24 years and −25.3 for 25–34 years), reflecting a tendency toward moderate endorsement. Respondents aged 35–44 years exhibited a polarized pattern of responses (*strongly agree* +18.5; *agree* −21.7). Individuals aged 45–54 predominantly endorsed *strongly disagree* (+24.5) and were underrepresented in strongly agree (−24.8), whereas those aged 55–64 years showed a fragmented response profile (*agree* +28.6; *strongly agree* −29.8). Participants aged 65 years and above most frequently endorsed *strongly agree* (Figure 2).

Taken together, these patterns suggest that age meaningfully shapes the degree and intensity of endorsement of daily survival-related food purchases, with younger adults exhibiting more moderate positions and older adults demonstrating stronger, more extreme agreement.

**Null Hypothesis H01.3.**  *There are no significant differences between education levels in terms of food purchases driven by survival needs (daily food).*

The adjusted Rao & Scott test did not identify significant differences in daily survival-related food purchases across educational levels. The resulting statistic, F [4.51; 4784.7] = 1.65, *p = 0.15*, indicates that individuals with varying educational backgrounds have a statistically comparable distribution of such purchases. Variation in educational attainment does not appear to account for differences in the likelihood of purchasing basic daily food items.

Descriptive analysis revealed an unbalanced distribution across the agreement categories of the Likert scale: responses with middle school, post-secondary, university, and postgraduate education levels were over-represented in the *agree* category (+122.3 for middle school) and in the *strongly agree* category (+73.5 for post-secondary; +11.6 for university; +0.5 for postgraduate), while underrepresentation occurred for *agree* (−53.7 for post-secondary; −31.2 for university; −13.5 for postgraduate) and *strongly agree* (−77.6 for middle school). Respondents with high school education tend to cluster around the *neutral* category, with the largest positive difference observed at the *neutral* level (+39.1) (Figure 2).

**Null Hypothesis H01.4.**  *There are no significant differences between marital status categories in terms of food purchases driven by survival needs (daily food).*

The Rao-Scott adjusted chi-square test revealed a significant association between marital status and food purchases motivated by survival needs. The analysis yielded F [2.76; 2923.62] = 6.85, *p = 0.0002*, indicating that the observed differences are unlikely to be due to chance. These results suggest that marital status is meaningfully related to patterns of daily food purchasing behavior.

Descriptive analyses of observed versus expected values highlighted distinct patterns in survival-driven food purchases across marital status. Married respondents were strongly represented in the *agree* category (+96.2) and underrepresented in neutral (−76.1), indicating that survival needs predominantly motivated their purchases. Single respondents showed the opposite pattern, with overrepresentation in *neutral* (+76.7) and underrepresentation in *agree* (−92), suggesting a lower influence of survival needs. Those in a relationship exhibited more variable responses, with slight underrepresentation in *agree* (−4.2) and overrepresentation in strongly agree (+4.2), reflecting heterogeneous attitudes toward survival-driven food purchases (Figure 2).

**Null Hypothesis H01.5.**  *There are no significant differences between residence categories in terms of food purchases driven by survival needs (daily food).*

The analysis produced F [1.97; 2088.56] =2.61, *p = 0.07*, indicating that while purchasing patterns vary somewhat by residence, these differences do not reach conventional levels of statistical significance. Thus, residence appears to be only weakly associated with daily food acquisition behavior, although minor trends may warrant further investigation.

Descriptive analyses revealed distinct patterns between rural and urban respondents regarding food as a survival necessity. Rural respondents were overrepresented in the *agree* category (+111.8) and slightly in *disagree* (+7.5), but underrepresented in *strongly agree* (−84.1), indicating moderate endorsement of food as a survival necessity. In contrast, urban respondents were overrepresented in *strongly agree* (+84.1) and *neutral* (+27.9), and underrepresented in *agree* (−111.8), reflecting a more polarized distribution with a tendency toward extreme or neutral responses (Figure 2).

**Null Hypothesis H01.6.**  *There are no significant differences between income categories in terms of food purchases driven by survival needs (daily food).*

The results of the applied statistical procedures indicated differences in daily survival-related food purchases across income categories. The analysis yielded F [4.69; 4974.7] = 1.84, *p = 0.10*, suggesting that while variation exists among income groups, it does not reach conventional levels of statistical significance. Accordingly, there is insufficient evidence to suggest a meaningful association between income and the likelihood of purchasing basic daily food items.

Descriptive statistics revealed distinct patterns of survival-driven food purchases across income groups. Very low-income households (below 2500 RON) were polarized between *neutral* (+36.2) and *strongly agree* (+15.4). Low-to-middle-income households (2501–3500 RON) clustered in *agree* (+65.3) and were underrepresented in *strongly agree* (−85), whereas middle-income households (3501–4500 RON) were overrepresented in *strongly agree* (+90). Households earning 4501–5500 RON leaned toward *neutral* (+14.9), and those in the 5501–6500 RON range were overrepresented in *agree* (+66.4) and underrepresented in *strongly agree* (−40.8) and *neutral* (−20.3). High-income households (over 6500 RON) showed only minor differences, slightly favoring *strongly agree* or *neutral* (Figure 2). These patterns suggest that, while survival needs remain relevant across all income levels, higher-income households exhibit reduced urgency in survival-driven food purchases.

### 3.2. The Need for Food Security (Food Stocks)

**Null Hypothesis H02.1.**  *There are no significant differences between gender categories in terms of food purchases driven by security needs (food stocks).*

Analysis indicated statistically significant differences in food purchases motivated by security needs (food stocks) across gender categories, F [2.71; 2875.26] = 4.77, *p* = 0.003. The findings indicate that men and women exhibit distinct patterns in food stockpiling behaviors, reflecting meaningful variation in this aspect of purchasing behavior.

**Figure 3:**
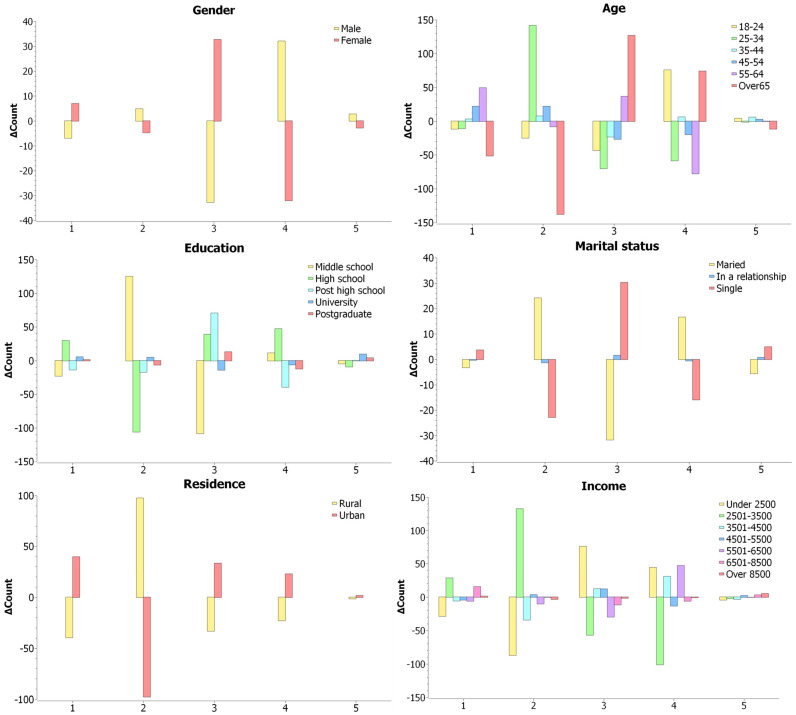
The difference between count and expected count for food purchases driven by security needs. Notes: 1 = strongly disagree; 2 = disagree, 3 = neutral, 4 = agree, 5 = strongly agree. ∆Count > 0 indicate overrepresentation and ∆Count < 0 indicate underrepresentation.

Descriptive analyses of observed versus expected values showed that men were overrepresented in the *agree* category (+118.3) and underrepresented in *disagree* (−155.9), highlighting a clear recognition of food security as a motivating factor in purchasing decisions. In contrast, women were overrepresented in *disagree* (+155.9), reflecting a more nuanced or pragmatic approach to food purchasing behavior (Figure 3).

**Null Hypothesis H02.2.**  *There are no significant differences between age categories in terms of food purchases driven by security needs (food stocks).*

The Rao & Scott-adjusted Pearson chi-square test indicated a highly significant effect of age on food purchases driven by security needs (food stocks), F [6.67; 7068.99] = 6.28, *p* = 4.04 × 10^−7^. Observed versus expected values highlighting clear age-related contrasts: young adults (18–24 years) strongly recognized the importance of food stockpiling (*agree* +76), whereas those aged 25–34 years placed less emphasis on preventive purchases (*disagree* +141.7). Middle-aged respondents (45–64 years) tended to downplay food security, with positive deviations in *disagree* (+22.1) and *strongly disagree* (+49.4), whereas respondents over 65 years showed a balanced approach, with overrepresentation in *neutral* (127.2) and *agree* (+74.4) (Figure 3). These patterns suggest distinct age-related differences in security-driven purchasing behavior.

**Null Hypothesis H02.3.**  *There are no significant differences between education levels in terms of food purchases driven by security needs (food stocks).*

The analysis indicated only a borderline association between education level and food purchases motivated by security needs (food stocks), F [4.05; 4290.38] = 2.33, *p = 0.05*, suggesting that educational differences do not translate into marked contrasts in stockpiling behavior. Nevertheless, the distribution of responses reveals several noteworthy tendencies.

Individuals with middle school education were concentrated in the *disagree* category (+125.1), indicating a comparatively low inclination toward viewing food reserves as necessary. High-school graduates exhibited a more diffuse pattern, with elevated frequencies in both *agree* (+47.1) and *strongly disagree* (+29.8), alongside a substantial deficit in *disagree* (−106.4), indicating a less uniform stance within this group (Figure 3). University and postgraduate respondents showed only minor deviations across categories, a pattern suggestive of more differentiated, but not polarized, perceptions of food security at higher educational levels.

**Null Hypothesis H02.4.**  *There are no significant differences between marital status categories in terms of food purchases driven by security needs (food stocks).*

The Rao & Scott-adjusted analysis revealed no significant differences across marital status categories in food purchases motivated by security needs. The analysis yielded F [2.54; 2697.93) = 0.66, *p* = 0.55, indicating that the observed variation does not exceed what would be expected under random sampling fluctuation. Marital status does not appear to be associated with distinct patterns of food stockpiling behavior.

Descriptive analyses showed that married respondents were overrepresented in *disagree* (+24.2) and *agree* (+16.6) but underrepresented in *neutral* (−31.8), indicating avoidance of moderate positions. Single respondents favored *neutral* (+30.3) and were underrepresented in *disagree* (−22.9) and *agree* (−16), reflecting a fragmented orientation. Those in a relationship were primarily overrepresented in *neutral*, suggesting a preference for a balanced approach to food security (Figure 3).

**Null Hypothesis H02.5.**  *There are no significant differences between residence categories in terms of food purchases driven by security needs (food stocks).*

Respondents appear to share similar concerns regarding food security, regardless of whether they reside in urban or rural areas, reflecting a relatively uniform approach to food stockpiling in response to uncertainties. Residence does not appear to influence food stockpiling behaviors. The results support this conclusion, yielding F [2.13; 2258] = 1.33, *p* = 0.26, indicating no statistically significant differences across the categories of residence.

Descriptive analyses showed that rural respondents were strongly concentrated in the *disagree* category (+97.8), with consistent underrepresentation across all other response options, indicating a limited emphasis on food security as a purchasing motive. Urban respondents displayed the opposite pattern, being markedly underrepresented in *disagree* (−97.8) and overrepresented in *agree* (+23), suggesting a clearer alignment between food purchases and security considerations (Figure 3).

**Null Hypothesis H02.6.**  *There are no significant differences between income categories in terms of food purchases driven by security needs (food stocks).*

In the literature, income is widely recognized as an important determinant of food security, as low-income households are typically more vulnerable to food insecurity [70,71,72,73,74,75]. However, access to alternative support sources and social protection policies may mitigate these effects, which sometimes explains the absence of clear and significant differences in survey-based studies [76].

The applied tests indicated no statistically significant differences in food purchases driven by security needs across income categories, (F [5.67; 6008.52] =1.99, *p* = 0.06). The observed patterns, however, highlight subtle contrasts: very low-income household (<2500 RON) gravitated toward *neutral* (+76.2) and *agree* (+44.6), reflecting moderate acknowledgment of food security, whereas low-to-middle incomes (2501–3500 RON) favored *disagree* (+132.8) and *strongly disagree* (+28.8), indicating lower emphasis on preventive purchases. Upper-middle-income households (5001–6500 RON) leaned toward *agree* (+47.1), while the highest-income groups (>8500 RON) showed slight overrepresentation in *strongly agree* (+5.2), suggesting planned, security-oriented behavior (Figure 3). Overall, these trends imply that income subtly influences the extent and manner in which food security motivates purchasing.

**For RQ2: Multinomial logistic regression model **

Given that the Likert scales measuring fundamental food needs are inherently ordinal, ordinal logistic regression is the natural first modeling choice, provided that predictors exert comparable effects across outcome categories—a condition known as proportional odds assumption [77]. Brant-Wald tests were applied to assess this assumption for the ordinal logistic regressions estimated for each fundamental food need using the *polr()* function from the *MASS* package (R, version 4.4.3) [78].

Because the proportional odds assumption was violated for security needs, we proceeded to the next viable modeling choice: multinomial logistic regression. Considering that the fundamental food needs are response variables with five outcome levels, multinomial logistic regression is an appropriate method for modeling the data. Analyses were conducted in R (version 4.4.3) [59,64] using the *multinom()* function in the *nnet* package (R, version 4.4.3), which is based on neural network algorithms [77,78].

Two weighted multinomial logistic regressions were performed—one for each fundamental food need (survival need and food security)—as a function of the explanatory variables: gender, age, education, income, residence, and marital status. This method estimates the odds of belonging to each outcome category of the response variable (fundamental food need) relative to a reference category. The category *strongly disagree* (the lowest point on the Likert scale) was selected as the reference.

The model evaluates the statistical significance (*p*-value) for each independent variable, where *p* < 0.05 indicates that the variable is a significant predictor with a meaningful effect on the relative odds of the response variable. For each significant predictor, Table 1 and Table 2 present the regression coefficients (β), standard errors (S.E.), Wald *z*-values, associated significance levels (*p*-values), odds ratios [Exp(β)], and confidence intervals [CI for Exp(β)].

For predictors with more than two categories, only the dominant linear contributions—denoted by the *.L* suffix—are reported, as higher-order terms (quadratic or beyond) had negligible influence. Regression coefficients (β) represent the change in the logarithm of relative odds associated with a one unit increase in the predictor. The exponential coefficients, Exp(β), express this effect as a multiplicative change in relative odds: values greater than one indicate increased odds, whereas values lower than one indicate decreased odds. For categorical predictors with multiple levels, the coefficients (β) and odds ratio [Exp(β)] corresponding to linear contrasts (denoted by the *.L* suffix) carry the same interpretation.

To evaluate model fit and overall explanatory power, McFadden’s, Cox & Snell’s, and Nagelkerke’s pseudo-R^2^ coefficients were calculated for both multinomial logistic regression models [67,68,69]. The results indicated that each model provided a good fit, effectively explaining the variability in the observed data.

As shown in Table 2, for the *survival need*, age has a statistically significant impact (*p* < 0.05) across all response categories relative to the reference category. Residence is relevant at the lower end of the Likert scale, education level at the middle levels, while marital status and income are significant at the upper levels of agreement. Gender exceeds the significance threshold for the *agree* and *disagree* categories, though only marginally, suggesting that gender is primarily relevant in the midrange of the Likert scale.

The coefficients obtained from the multinomial logistic regression allow estimation of how the relative odds of belonging to a specific response category (compared to the reference category) change when a predictor varies while all other predictors are held constant. For instance, an increase in education level significantly decreases the relative odds of being in the *neutral* or *agree* categories compared to *strongly disagree*. Conversely, older respondents are more likely to belong to the *strongly agree*, *agree*, or *neutral* categories, and less likely to be in the *disagree* category. Being male increases the odds of being in the *agree* category, whereas higher income increases the odds of being in *strongly agree* relative to *strongly disagree* by a factor of approximately 18. Similarly, being in a relationship or married raises the odds of belonging to the *agree* category by a factor of 4.8, and to *strongly agree* by a factor of 3.2.

To assess the model fit and explanatory power, the McFadden’s [67], Cox & Snell’s [68], and Nagelkerke’s [69] pseudo-R^2^ coefficients were calculated for the multinomial logistic regression of survival need. The obtained values, McFadden’s R^2^ ≅ 0.385, Cox & Snell’s R^2^ ≅ 0.55 and Nagelkerke’s R^2^ ≅ 0.629, indicate good model fit and explanatory power. The Nagelkerke’s value suggests that the model explains approximately 63% of the variance in the data, reflecting excellent explanatory power.

The full model incorporates all sociodemographic variables as explanatory factors. In principle, more parsimonious specifications may be considered to improve explanatory or predictive performance by excluding one or more demographic variables. Such models can be compared using the Akaike Information Criterion (AIC) [79] and the Bayesian Information Criterion [80]. For the full model, the obtained values were AIC ≅ 2387.9 and BIC ≅ 2825.2. By comparison, a model excluding income yielded AIC ≅ 2581.2 and BIC ≅ 2887.3, while a model including only gender, marital status, and education resulted in AIC ≅ 2872 and BIC ≅ 3046.9. These results indicate that the full model offers superior explanatory and predictive performance, as lower AIC and BIC values denote better model fit. Given its strong explanatory capacity, further examination of more parsimonious model specifications is beyond the scope of the present study.

For the food security need, a similar multinomial logistic regression was performed, and the statistically significant predictors are presented in Table 3. The pseudo-R^2^ coefficients (McFadden’s R^2^ ≅ 0.434, Cox & Snell’s R^2^ ≅ 0.683, and Nagelkerke’s R^2^ ≅ 0.735) indicate that the model explains over 73% of the variance in the data, suggesting a very good model fit and strong explanatory power. Given this high explanatory capacity, the exploration of more parsimonious models is beyond the scope of this article.

As shown in Table 3, education level and residence are significant predictors across all response categories relative to the reference category (*strongly disagree*). Gender is statistically significant only for the *disagree* category, while marital status is significant only for the *agree* category. Age emerges as a good predictor for the *neutral* and *strongly agree* categories.

The results indicate that higher education levels substantially decrease the relative odds of belonging to the *disagree* and *agree* categories, while increasing the likelihood of being in the *neutral* and *strongly agree* categories by factors of approximately 2.9 and 29.6, respectively. Urban residence reduces the likelihood of belonging to any category other than *strongly disagree*. Being male decreases the odds of being in the *disagree* category relative to the reference category by 79%. Older respondents are less likely to fall into the *strongly agree* category but are 6.6 times more likely to belong to the *neutral* category. Finally, being in a relationship or married significantly reduces the odds of being in the *agree* category compared to the *strongly disagree* reference group.

## 4. Discussion

Food security is predominantly analyzed in crisis contexts—such as pandemics, supply chain disruptions, or economic uncertainties—and is mainly associated with stockpiling behaviors generated by external pressures [6,7,8,9,57]. The present study conceptualizes food security from a structural and relatively stable perspective, focusing on purchases made to build reserves within the routine of everyday consumption, in the absence of immediate disruptive events. Nevertheless, considering recent crisis-related experiences and supply chain disruptions during the COVID-19 pandemic, it cannot be excluded that certain preventive behaviors continue to reflect patterns formed under conditions of instability.

Romania has maintained relatively stable living standards in recent years, with purchasing power remaining steady and access to consumer goods broadly available. According to the National Institute of Statistics, Romania’s price levels for consumer goods and services were among the lowest in the EU in 2023—approximately 40% below the EU average—helping to sustain purchasing power despite income disparities [81]. At the same time, GDP per capita measured in Purchasing Power Standards (PPS) has steadily increased, reflecting improvements in living conditions and consumption capacity [82]. This combination of rising incomes and comparatively lower prices has ensured easier household access to a wide range of products, from everyday food items to durable goods. As a result, the Romanian consumer market has become more resilient, with stable purchasing power and improved product availability supporting a relatively consistent standard of living.

Overall, the results highlight significant differences among sociodemographic categories in their relationship to the fundamental needs of survival and security, as conceptualized within Maslow’s hierarchy of needs, in the context of food consumption. This study forms part of a broader research project investigating the relationships between sociodemographic characteristics and multiple dimensions of food consumption behavior, including health status, consumption habits (e.g., dehydrated or derivative products), and the criteria guiding food purchases—such as promotions, price—quality ratios, social influences, and product familiarity [23,58].

### 4.1. Gender

The results of the study show that food purchases driven by survival needs (daily food) do not differ significantly between men and women, confirming the universal character of this physiological dimension, which is less influenced by sociodemographic factors. Similar convergences have been reported in the recent literature, which indicates that immediate access to food exhibits limited variation by gender [83].

In contrast, gender differences were statistically significant in the case of food purchases driven by security needs (food stocks). Men tend to attach greater importance to food storage, an attitude that may reflect an orientation toward control and resource stability, closely linked to risk perception. This pattern is supported by previous research highlighting that men may adopt resource control strategies in situations of food vulnerability [84,85]. Conversely, women are more likely to adopt immediate and pragmatic food management strategies [74,75]. As they are more frequently responsible for organizing daily food resources, women tend to favor short-term management practices such as consumption optimization, product prioritization, and budget adjustment [86]. In this context, stockpiling may be perceived as inefficient or impractical, thereby reducing its prioritization.

Food needs are not solely biological but are also shaped by perceptions, emotions, risk, social norms, and broader economic and social contexts. Although basic needs provide a general conceptual framework, gender-related variations are more effectively explained by risk perception models [85,87,88,89], theories of gender roles in household economics [74,90], and behavioral economics approaches [91,92].

The multinomial logistic regression for the *need to survive* further indicated that gender is a significant predictor for the *disagree* and *agree* categories relative to the reference category. It is important to note that multinomial logistic regression offers a more nuanced analytical perspective than chi-square tests, as it accounts for the simultaneous influence of multiple variables. While chi-square tests identify associations without controlling for confounding factors, logistic regression adjusts for these effects, thereby clarifying the independent contribution of each predictor.

### 4.2. Age

The analysis of differences across age groups revealed distinct patterns in food purchases motivated by the basic needs of survival and security, as defined within Maslow’s hierarchy. In interpreting these findings, it was considered that food-related decisions are shaped not only by physiological needs but also by the interaction between external factors (such as supply, price, and accessibility) and internal factors (including beliefs, norms, and risk perception).

Young respondents (18–34 years) are less likely to purchase food primarily to satisfy basic needs. This tendency may be explained by the life stage characteristics of this group, which involve lower pressure related to food provision and greater freedom to explore food options. Consequently, preferences, experimentation, and convenience often guide food choices in this age group [93,94,95,96,97].

Individuals aged 45–54 and 55–64 years tend to occupy a more neutral position. Within the logic of Maslow’s hierarchy, this segment appears to balance the need for daily food with the need for security, adopting adaptive but not excessive purchasing behaviors. For this group, external contextual factors exert a more direct influence on food-related decisions [98,99].

Among respondents aged 65 years and over, a strong orientation toward food security needs is observed. This pattern is consistent with Maslow’s theory, which suggests that once physiological needs are stably satisfied, the importance of safety needs increases. Life experiences related to scarcity or crisis, heightened perceived vulnerability, and the economic prudence often associated with older age contribute to more pronounced preventive behaviors [100,101].

Overall, the results indicate that age significantly shapes individuals’ relationships with basic dietary needs, with a transition from preference-oriented consumption in younger populations to consumption anchored in security, stability, and prevention in older age groups.

The multinomial logistic regression analysis confirmed that age is a significant predictor across all response categories for food purchases driven by survival needs. Increasing age markedly decreases the odds of belonging to the *disagree* category, while substantially increasing the odds of belonging to the *neutral*, *agree*, or *strongly agree* categories relative to the *strongly disagree* reference category.

For food security, age emerged as a significant predictor for the *neutral* and *strongly agree* categories, with increasing age substantially raising the odds of belonging to the *neutral* category relative to the reference category.

### 4.3. Education

The analysis of the distribution of responses (observed and theoretically expected) highlighted variations across educational levels in the reporting of food purchases driven by survival needs. However, these differences are not statistically significant, reinforcing the fundamental and universal character of daily food needs, as postulated by Maslow’s model. In contrast, the differences observed in food purchases driven by security needs are only marginally statistically significant. The results suggest that individuals with higher levels of education are more likely to interpret food purchasing behavior through a food security lens, whereas those with lower educational attainment tend to reject this perspective. This pattern is consistent with findings reported in the literature [87,102,103].

These observations align with previous research indicating that individuals with higher education levels tend to be better informed about crisis preparedness and, consequently, place greater emphasis on stockpiling [3]. Moreover, educational attainment is often correlated with income, providing higher-educated individuals with greater financial capacity to support preventive behaviors [104]. By contrast, individuals with lower levels of education may prioritize immediate purchases due to limited economic flexibility for stockpiling.

In the multinomial logistic regression analysis, education emerged as a significant predictor for the *neutral* category in food purchases driven by survival needs, with increasing educational attainment substantially decreasing the odds of belonging to this category. For food security needs, education was a significant predictor across all response categories, with higher education levels increasing the odds of belonging to the *neutral* category relative to the *strongly disagree* reference category.

### 4.4. Marital Status

The analysis highlights that marital status significantly influences food purchases motivated by survival needs. Married individuals pay greater attention to meeting basic needs, whereas single respondents prioritize these needs to a lesser extent. Individuals who are in a relationship exhibit a more varied, intermediate orientation. These differences can be explained by varying levels of involvement in the food purchasing process: family responsibilities and household structure lead married individuals to prioritize fundamental needs more clearly, whereas single individuals’ perceptions and justifications for food purchases may be shaped by autonomy and limited social support [105,106].

In contrast, food purchases motivated by stockpiling appear to represent a common concern across all three marital status categories, indicating that food storage and preventive behaviors are more generalized and less dependent on family structure. This finding aligns with the existing literature, showing that, in contexts of uncertainty or instability, the tendency to stockpile food and ensure constant access becomes a collective behavior that is less influenced by marital status [36].

Overall, the results suggest that family responsibilities play a role in prioritizing survival needs, whereas concern for food security remains relatively constant across marital status categories. This indicates that both external factors (such as supply, price, affordability, and uncertainty) and internal factors (including risk perception and social norms) contribute to shaping food-related behaviors, without any single factor acting as an exclusive determinant.

The multinomial logistic regression analysis further indicates that marital status is a significant predictor for the relative odds of belonging to the *agree* and *strongly agree* categories for survival-driven food purchases, as well as of the *agree* category for food purchases driven by food security needs.

### 4.5. Residence

The comparative analysis between urban and rural areas indicates the presence of subtle variations in how respondents report food purchases driven by both survival and security needs. However, in neither case do these differences reach the threshold of statistical significance.

These variations between respondents from the two residential contexts are likely to arise from the interaction between external factors (such as product accessibility, diversity of supply, and income levels) and internal factors (including risk perception, social norms, and individual preferences). Concern for meeting food needs—both daily consumption and stockpiling—appears to be more pronounced in urban areas.

This pattern may be associated with the need for control characteristic of urban lifestyles, where stockpiling can reduce the frequency of store visits and alleviate anxieties related to food availability and affordability. In contrast, access to self-produced resources (e.g., home gardens, preserved foods, or livestock) provides rural households with greater autonomy and flexibility in food-related decision-making [107,108,109].

From a predictive modeling perspective, residence exhibits a pattern similar to that of education level: it emerges as a significant predictor across all response categories for food driven by security needs. For survival-driven food purchases, residence is a significant predictor only for the relative odds of belonging to the *disagree* category relative to the *strongly disagree* reference category.

### 4.6. Income

The results indicate that purchasing food for daily consumption remains fundamental and universal, regardless of income level, supporting Maslow’s model. Although differences between income categories are not statistically significant, the descriptive profiles reveal noteworthy trends. As income increases, responses shift from the vulnerability and polarization observed among low-income groups toward relative stability in higher-income groups, where the perceived pressure to secure survival through food diminishes but does not entirely disappear [110,111,112,113].

The relationship between income and food purchases for stockpiling is similarly nuanced. Although respondents from very low-income households acknowledge the importance of food security, limited resources constrain sustained engagement in preventive behaviors such as food storage. By contrast, low- and middle-income households tend to reject preventive measures, suggesting that food consumption is primarily oriented toward meeting immediate needs rather than planning for long-term reserves. This orientation can likely be explained by financial constraints, whereby disposable income is sufficient to cover current needs but insufficient to support preventive behaviors. As income increases further, a more balanced pattern emerges. Strongly defined positions are observed among very high-income households, with respondents predominantly selecting strongly agree. This finding suggests that food security is either actively ensured through preventive behaviors or perceived as less pressing due to easy access to resources associated with higher levels of household economic stability.

These findings align with the literature, which identifies income as a key determinant of food security, although its influence is moderated by contextual factors such as social support networks, policy interventions, and access to food distribution infrastructure [70,71,72,73,74,75,76].

Multinomial logistic regression analyses further indicates that income is a significant predictor only for the *strongly agree* category in survival-driven food purchases, with higher income increasing the odds of belonging to this category relative to the *strongly disagree* reference category.

Overall, the analysis of socio-demographic differences in food consumption demonstrates that motivations are heterogeneous and shaped by factors such as age, education, marital status, and gender [114]. This heterogeneity highlights the declining effectiveness of uniform marketing strategies [115,116,117,118]. In contrast, manufacturers and retailers that identify and address the specific characteristics of each consumer segment can foster stronger engagement and enhance brand loyalty [93,94,119,120,121]. Beyond its theoretical contributions, this study also offers practical insights by providing benchmarks for the development of marketing strategies that address not only basic food needs but also higher-level needs related to consumer consideration and self-realization [122,123,124,125].

### 4.7. Potential Applications of the Results

The observed socio-demographic differences indicate that, while basic food needs remain universal, the ways in which individuals relate to survival and food security vary according to resources, accessibility, and risk perception. These insights provide practical implications for economic operators and policy design.

Product segmentation based on household resources can improve the alignment of supply with consumer needs. Low-income consumers may benefit from essential and affordable products, whereas higher-income and higher-education groups may respond better to storage-oriented packages or premium options tailored to preventive purchasing behaviors.

Improving access and distribution is particularly relevant in rural areas. Expanding product availability and maintaining stable stocks can reduce vulnerability in contexts of uncertainty, supporting both daily consumption and preventive stockpiling strategies.

Targeted communication strategies can enhance engagement and understanding. Messages emphasizing safety, continuity, and reliability may be more effective for vulnerable groups, such as the elderly or low-income households, while highly educated consumers may respond better to detailed, transparent, and evidence-based information regarding product quality, supply stability, and stockpiling recommendations.

Risk perception management is another key consideration. Ensuring transparency in supply chain stability can support preventive purchasing behaviors without triggering panic-driven stockpiling, allowing consumers to make informed decisions in the context of uncertainty.

Finally, inclusive initiatives aimed at supporting vulnerable households can strengthen food security. Tailored loyalty programs, price-guarantee products, or strategic partnerships can improve access and reduce disparities, reinforcing the ability of households to meet both survival and security needs effectively.

## 5. Conclusions

The exploratory nature of this research provided the flexibility to identify socio-demographic predictors and test hypotheses concerning the roles of gender, age, educational level, marital status, residence, and income in explaining variations in food purchasing behavior.

The results indicate that the relationship between food purchases and fundamental needs varies significantly across socio-demographic groups. Men tend to associate food shopping more directly with protection and stability, whereas women are less likely to view this motivation as central, potentially emphasizing lifestyle, health, and family cohesion instead. Young adults (18–34) are less motivated by survival and food security when buying food, focusing more on preferences and convenience. In contrast, those over 65 years of age are more concerned about these basic needs, while individuals aged 45–64 take a balanced, pragmatic approach.

Regarding education, although differences are only marginally statistically significant, patterns suggest that higher educational attainment may enhance the capacity to adopt preventive behaviors in food purchasing. Marital status also influences the prioritization of survival needs: married individuals view these needs as central, single individuals less so, and those in a relationship adopt an intermediate approach, reflecting variations in family responsibilities, household structure, and social support.

A key contribution of this study is demonstrating that food purchases driven by fundamental needs can be effectively modeled using multinomial logistic regression. This approach allowed the identification of significant socio-demographic predictors and the estimation of their effects on the relative odds of belonging to each response category relative to the reference category.

Future research should explore how socio-demographic variables—such as age, income, education, and place of residence—correlate with higher-order needs in Maslow’s hierarchy, including belonging, esteem, and self-actualization. Building on the findings of the present study, this line of research aims to provide a more comprehensive understanding of food acquisition behaviors, encompassing both physiological and psychological motivations.

## Figures and Tables

**Table 1:** Sociodemographic characteristics of the weighted survey design.

Characteristics	Share in the Sample	Percentage (%)
Gender	Female	40
	Male	60
Age (years)	18–24	11.6
	25–34	15.1
	35–44	7.1
	45–54	11.6
	55–64	13.8
	Over 65	40.8
Marital status	Single	13.8
	In a relationship	0.7
	Married	85.5
	Middle school education	17.3
Education level	High school education	57.2
	Post-secondary education	13.5
	University education	6.85
	Postgraduate education	5.1
Household monthly net income (RON)	Under 2500	25
	2501–3500	26.1
	3501–4500	26.9
	4501–5500	3.7
	5501–6500	12.3
	6501–8500	2.9
	Over 8500	3.1
Residence	Urban	64.1
	Rural	35.9

**Table 2:** Regression coefficients and corresponding statistics for the significant predictors of the *survival need*.

	β	S.E.	Wald z	Sig.	Exp(β)	95% C.I. for Exp(β)	
						Lower	Upper
*Disagree* relative to the reference category							
**Gender**							
*Male*	1.938	0.975	1.988	0.047	6.941	1.028	46.886
**Age**							
*Age.L*	−9.388	0.804	−11.670	0.000	$8.3\times 10^{-5}$	$1.7\times 10^{-5}$	$4\times 10^{-4}$
**Residence**							
*Urban*	−2.9951	0.931	−3.216	0.001	0.050	0.008	0.310
*neutral* relative to the reference category							
**Age**							
*Age.L*	9.689	0.625	15.493	0.000	16,145.01	4738.955	55,003.95
**Education Level**							
*Education level.L*	−1.693	0.465	−3.639	$2.7\times 10^{-4}$	0.184	0.074	0.458
*agree* relative to the reference category							
**Gender**							
*Male*	1.438	0.434	3.316	$9.1\times 10^{-4}$	4.211	1.800	9.851
**Age**							
*Age.L*	8.299	0.604	13.736	0.000	4021.488	1230.484	13,143.10
**Marital Status**							
*Marital status.L*	1.573	0.409	3.845	0.0001	4.819	2.162	10.743
**Education Level**							
*Education level.L*	−13.438	0.482	−27.879	0.000	$1.4\times 10^{-6}$	5.6 $\times 10^{-5}$	3.7 $\times 10^{-6}$
*stronglyagree* relative to the reference category							
**Age**							
*Age.L*	7.04402	0.608566	11.57478	0.000	1145.990	347.671	3777.407
**Marital Status**							
*Marital status.L*	1.176305	0.431468	2.72628	0.006	3.242	1.392	7.553
**Income**							
*Income.L*	2.90569	1.14225	2.54383	0.011	18.278	1.948	171.479

**Table 3:** Regression coefficients and corresponding statistics for the significant predictors of the *food security need*.

	β	S.E.	Wald z	Sig.	Exp(β)	95% C.I. for Exp(β)	
						Lower	Upper
*Disagree* relative to the reference category							
**Gender**							
*Male*	−1.545	0.321	−4.814	$1.5\times 10^{-6}$	0.213	0.114	0.400
**Education Level**							
*Education level.L*	−10.421	0.391	−26.638	0.000	$3.0\times 10^{-5}$	$1.4\times 10^{-5}$	$6.4\times 10^{-5}$
**Residence**							
*Urban*	−2.281	0.469	−4.863	$1.2\times 10^{-6}$	0.102	0.041	0.256
*neutral* relative to the reference category							
**Age**							
*Age.L*	1.892	0.654	2.895	0.004	6.632	1.842	23.876
**Education Level**							
*Education level.L*	1.066	0.333	3.205	0.001	2.903	1.513	5.570
**Residence**							
*Urban*	−3.960	0.439	−9.024	0.000	0.019	0.008	0.045
*agree* relative to the reference category							
**Marital Status**							
*Marital status.L*	1.668	0.355	4.696	$2.7\times 10^{-6}$	5.302	2.643	10.637
**Education Level**							
*Education level.L*	−9.150	0.380	−24.060	0.000	$1.1\times 10^{-4}$	5.0 $\times 10^{-5}$	2.2 $\times 10^{-4}$
**Residence**							
*Urban*	−3.034	0.474	−6.406	$1.5\times 10^{-10}$	0.048	0.019	0.122
*strongly agree* relative to the reference category							
**Age**							
*Age.L*	−12.569	0.654	−19.216	0.000	$3.5\times 10^{-6}$	$9.7\times 10^{-7}$	$1.3\times 10^{-5}$
**Education Level**							
*Education level.L*	3.389	0.435	7.798	6.2 $\times 10^{-15}$	29.648	12.648	69.494
**Residence**							
*Urban*	−2.302	0.608	−3.788	$1.5\times 10^{-4}$	0.100	0.030	0.329

## Data Availability

The data presented in this study are available on request from the corresponding authors. The data are not publicly available due to privacy restrictions.
